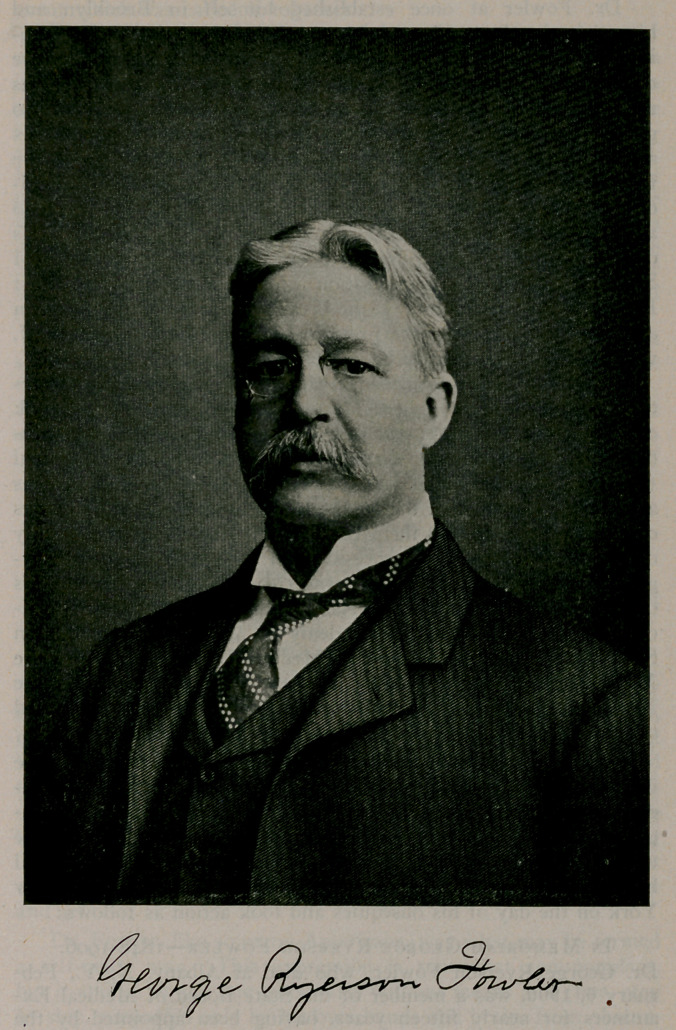# George Ryerson Fowler

**Published:** 1906-02

**Authors:** 


**A Monthly Review of Medicine and Surgery.**

*EDITOR:*

WILLIAM WARREN POTTER, M. D.

All communications, whether of a literary or business nature, books for review and exchanges, should be addressed to the editor 284 FRANKLIN ST., BUFFALO, N. Y.

VOL. LXI. FEBRUARY, 1906. No. 7

### George Ryerson Fowler.

BORN DECEMBER 25, 1848. DIED FEBRUARY 5, 1906.

THE medical profession of the entire country was painfully shocked when the wires flashed the sad news of the death of that eminent surgeon and distinguished citizen, Dr. George Ryerson Fowler, of Brooklyn. He had led such a busy life, and was in the midst of his greatest usefulness when the summons came, hence it seemed unusually difficult to believe it could be true.

The circumstances of Dr. Fowler's illness and death were painful in the extreme. He had been for four years a member of the committee of conference having in charge negotiations for the union of the two state medical organisations; he was a member and secretary of the centennial celebration committee of the society; a member of the State Board of Medical Examiners; and a member of the committee of the council of the Medical Society of the County of Kings, which had business with the newly organised state society relating to the property questions involved in the amalgamation. Each and all of the duties pertaining to these several offices made his presence at Albany important during the Centennial meeting of the Medical Society of the State of New York. He left home Saturday evening, January 27, and arrived at Albany the next morning, his purpose being to do some work during the two days previous to the meeting.

On the train Saturday night he fell ill and on his arrival at the Hotel Ten Eyck next morning he sent for Dr. Neuman, of Albany, who was his constant attendant for the next ten days. The serious nature of Dr. Fowler's illness became apparent to Dr. Neuman at once and he summoned Dr. Vander Veer in consultation. Subsequently, Dr. Bristow, of Brooklyn, was called, who came accompanied by Mrs. Fowler and son, Dr. Russell S. Fowler. Previous to their arrival a diagnosis of appendicitis had been made, which now was confirmed, whereupon the patient was removed immediately to the Albany hospital and prepared for operation. At eight o'clock Monday evening, January 29, Drs. Vander Veer and Macdonald removed a gangrenous appendix containing an enterolith. Three days afterward an enterostomy was done, under local analgesia, to relieve intestinal distension which had become distressful if not alarming. It is not our purpose to record the clinical history in detail but to touch upon its salient features. It is proper, however, to remark that during the earlier part of his illness Dr. Fowler was visited by Drs. McMurtry, Elsner, Bryant, Keen, and possibly some others, but Drs. Vander Veer, Macdonald, Bristow, and Neuman constituted the regular staff. These names are so well known to the professional world that everyone will understand how completely the art and science of medicine and surgery was exhausted in ministering to the distinguished patient.

From Thursday, when the enterostomy was made, onward for five or six days it was a battle for life. A skilful staff of surgeons and physicians were doing everything in their power to aid an intelligent patient who was making a brave struggle for existence against what proved to be overwhelming odds. The lines were compactly formed and moved forward and backward during these anxious days, but a steady recession was observed by the attending staff, each twenty-four hours showing a loss of vantage. Finally, at eight o'clock and fifteen minutes, Tuesday evening, February 6, 1906, the Son of man came and took away the spirit of George Ryerson Fowler, and he was at peace!

---

George Ryerson Fowler, son of Thomas W. and Sarah J. (Carmen) Fowler, was born in Brooklyn December 25, 1848. Soon after his birth, the family removed to Jamaica L. I., where he received his earlier education in the public schools. At the age of 14 he left school and home without permission, for the purpose of enlisting as a drummer-boy for service in the Civil War. At New York he was overtaken, brought back, and restored to his studies. A year or more later he became a telegrapher in the office of the Jamacia railway, and during an accident which happened soon afterward manifested such interest in the work of the surgeons that it is believed his resolve was then made to become a surgeon himself; at all events, in the course of time he entered the office of a surgeon as a student of medicine. He further pursued his studies at Bellevue Hospital Medical

George Ryerson Fowler

College, from which he received his doctorate degree in February 1871.

Dr. Fowler at once established himself in Brooklyn and his professional practice developed with almost phenomenal rapidity. He was appointed very soon a member of the staff of the central dispensary, serving in this capacity for two years. It was recognised soon that his skill as a surgeon amounted almost to genius, and his services were eagerly sought by hospitals, both as an attending and visiting surgeon. In 1877 he became one of the founders and the first secretary of the Brooklyn Anatomical Society and two years later he was chosen its president. He was also associate editor of the society's publication, then called Annals of the Anatomical and Surgical Society and later the Annals of Surgery. Upon the organisation of the Bushwick and East Brooklyn Dispensary in 1878 he became its first visiting surgeon and until 1887 he was the presiding officer of its medical staff, when he was appointed to be consulting surgeon.

In 1883 he became surgeon-in-chief to the department of fractures and dislocations in St. Mary's Hospital, and later he took charge of the entire general surgical department of that institution. He also became surgeon to the Methodist Episcopal (Seney) Hospital, which was founded in 1887. Dr. Fowler was a prominent member of the leading medical and surgical societies of this country, among them the Medical Society of the County of Kings, of which he was president in 1886; the American Surgical Association, of which he was treasurer at the time of his death; the American Medical Association, the New York Surgical Society, the New York Academy of Medicine, the Brooklyn Surgical Society, the Society of Medical Jurisprudence and the Medical Society of the State of New York. The Relief, the Norwegian and the Eastern District hospitals were also included among the institutions of which he was consulting surgeon.

When state examinations for license to practise medicine were instituted in 1891, Dr. Fowler, upon the nomination of the Medical Society of the State of New York, was appointed by the regents of the University, a state medical examiner. He chose the topic of surgery, which position he filled from that time until his death. The board met at the office of the secretary in New York on the day of his obsequies and took action as follows:

In Memoriam George Ryerson Fowler—1848-1906.

Dr. George Ryerson Fowler, who died at Albany, N. Y., February 6, 1906, was a member of the State Board of Medical Examiners for nearly fifteen years, having been appointed by the Regents of the University of the State of New York, March 11, 1891.

The board convened in special session this ninth day of February 1906, desires to express its profound sorrow because of the sad event which has called it together..

Dr. Fowler enjoyed the personal friendship of each and every one of the members, and we all feel a grevious personal affliction in his demise. He was so amiable in disposition, so cheerful in manner and, withal, so earnest in his work that the relations he sustained to us were more than official,—they were those of affection, and we each mourn him as a loved and loving friend. He was an indefatigable worker, a man of sterling integrity of purpose, whose shibboleth was fidelity to duty and loyalty to the interests confided to his care. Not alone is his loss one of serious import to his family circle and to his intimate friends, but the State suffers irreparably in losing the services of a public officer who had an eye only to right, equity, and progressiveness in all his acts.

Dr. Fowler's eulogium cannot be written in proper phrase or in sufficient detail at this time, but the foregoing expresses in outline the feeling and belief of his colleagues on the board.

Resolved: that this minute be spread upon the records and that an engrossed copy be transmitted to Mrs. Fowler.

William Warren Potter, M.D., President.
Maurice J. Lewi, M.D., Secretary.
William S. Ely, M.D.
Eugene Beach, M.D.
J. P. Creveling, M.D.
A. Walter Suiter, M.D.
Howard J. Rogers, LL.D., For the Department of Education.

---

The career of this interesting man was remarkable in many respects. Born in comparative penury he rose by his own energy, courage, and unflagging toil to a position, if not of affluence, at least of ease, with every comfort around him that a cultivated taste supported by an ample purse could wish or supply. In addition to his town house, filled with antiques and curios which he had gathered from all quarters of the globe, his country place at Glen Cove, known as "Crescent Beach-on-Sound," was a most delightful summer home, surrounded by every accessory of nature and art.

Dr. Fowler, like so many Americans, was what may be termed a self-made man. This expression is somewhat commonplace, but it applies to any one who starts without means and rises, step by step, supported only by his own hands and brain, from obscurity to fame, from poverty to ease, from ignorance to knowledge. Such is true of Dr. Fowler's singularly successful career. He became one of the most noted surgeons of his day, in this land of famous surgeons. As an operator he was noted for his simplicity of technic and for his narrow margin of fatalities. No surgical disease or injury was too difficult or complicated for him to undertake, and none in which he could not see a ray of hope, no matter how discouraging the outlook. Thus, he often wrung victory from the face of defeat, and saved many a patient that a less courageous or hopeful method would have lost.

Dr. Fowler, too, was an author of distinction. His contributions have been many and valuable, both in monographs and more enlarged works. We have inspected a list comprising nearly one hundred of the former, all of scientific value and some of great importance; while of the latter, his treatise on appendicitis is known throughout the world, having been translated into foreign languages, as one of the most valuable contributions upon that subject.

During the last twelve years of his life, Dr. Fowler had been engaged in preparing a treatise on general surgery which he intended should become a textbook for students and practitioners of medicine. The work is completed with the exception of the index, which latter he expected to finish while in Albany; but the cruel irony of fate stood between him and his long cherished hopes. A brief announcement of this work appeared in this JOURNAL for January.

Dr. Fowler was a frequent attendent upon local, state and national medical societies. He was a ready debater, his clear sonorous voice possessing carrying qualities that made him a pleasant speaker even before a large audience. His sunny countenance, sweet temper, and agreeable presence made him welcome in social circles. He was a wit of rare gifts, a raconteur of charming method, a well-equipped man, polished by travel and association with his fellow-men, so useful in the community, and so beloved by his friends and especially by a devoted, loving family, that it is painful to dwell upon the sadness caused by his removal from the scenes of his activity and from an environment that seemed so nearly perfect.

Dr. Fowler, early in professional life, displayed an aptitude for military service in the medical corps. Allusion has already been made to his ineffectual attempt to enlist during the Civil War. His first military service was as assistant surgeon, with the rank of captain, in the Fourteenth Regiment of Brooklyn. Nine years later he became surgeon of the regiment with the rank of major, and on October 5, 1886 he was appointed surgeon of the Second Brigade of Brooklyn. On April 2, 1898, his rank was advanced to that of lieutenant colonel. Of more recent date was his appointment as surgeon, with the grade of colonel, on the staff of General Roe, commander of the national guard of the state. He was made Brigadier General by brevet after 25 years service in the National Guard.

Dr. Fowler had made a careful study of hospital work as practised in the field during the Turko-Grecian war. He was, therefore, equipped with much personal experience when, at the time of the Spanish-American war, he was appointed to be chief surgeon of United States volunteers. He served from July 1, 1898, until January 31, 1899, in the seventh army corps commanded by General Fitzhugh Lee. He was assigned to duty as medical inspector, consulting surgeon, and chief of the operating staff. and performed duty in Florida and Cuba. During his service in Havana he did much to impress upon the United States authorities the need of a thorough system of sanitation in Cuba. His army field work was one of the most valuable services he ever renderd to th sciences of medicine and surgry.

In 1873, Dr. Fowler married Louise Rachel, youngest daughter of James Wells, of Norristown, Pa. Four children were born of this union, three of whom with Mrs. Fowler survive,—Russell S., a practising physician in Brooklyn; Florence G., a graduate of the Packer Collegiate Institute, 1898; and Royal H., a student of medicine at Cornell University. The sons will carry on the work of the father so suddenly interrupted.

The obsequies, consisting of the beautiful Episcopal service, were held Friday, February 9, at the church of the Messiah, Rev. Dr. Saint Clair Hester officiating, and the interment was at Greenwood. Owing to a heavy snow which covered the ground the military escort contemplated was omitted, and the orders to the Fourteenth Regiment, N. Y. N. G., were countermanded. The officers, however, attended in a body. The chancel of the church, a commodious one, was banked with flowers, and the capacity of the edifice was taxed even to overflowing, not all who desired being able to enter.

The honorary civilian pallbearers were: Dr. Joseph D. Bryant, New York; Dr. Henry L. Elsner, Syracuse; Dr. Abraham Jacobi, New York; Dr. Albert Vander Veer, Albany; Dr. Lewis S. Pilcher, Brooklyn; Dr. A. T. Bristow, Brooklyn; Dr. Charles A. Dana, New York; Dr. L. H. Neuman, Albany; Dr. William Warren Potter, Buffalo; Dr. Maurice J. Lewi, New York; Dr. A. Walter Suiter, Herkimer; Dr. Eugene Beach, Gloversville and Dr. Willis B. Gifford, Attica.

Also the Council of the Medical Society of the County of Kings, composed as follows: President, William Francis Campbell; Vice-President, Dr. Glentworth R. Butler; secretary, Dr. John A. Lee; assistant secretary, Dr. William A. Jewett; treasurer, Dr. O. A. Gordon; assistant treasurer, Dr. John R. Stivers; directing librarian, Dr. James T. Warbasse. Censors: Dr. J. C. Bierwirth, Dr. Henry G. Webster, Dr. Ralph H. Pomeroy, Dr. J. R. Kevin, and Dr. William C.Woolsey. Trustees: Dr. James W. Fleming, Dr. William Browning, Dr. Henry A. Fairbairn, Dr. Charles N. Cox, and Dr. John E. Shepherd.

The honorary military pallbearers, all members of the National Guard of New York, were: Major General Charles F. Roe, Brigadier General James McLeer, Colonel Nathaniel B. Thurston, Lieutenant Colonel George A. Wingate, Lieutenant Colonel James Wray Cleveland, Lieutenant Colonel William W. Ladd, Lieutenant Colonel Gilford Hurry, Lieutenant Colonel John N. Stearns, Jr., Major Frederick T. Leigh, Major John B. Holland, and Major Robert Kelly Prentice.

## Figures and Tables

**Figure f1:**